# Analysis of human synovial and bone marrow mesenchymal stem cells in relation to heat-inactivation of autologous and fetal bovine serums

**DOI:** 10.1186/1471-2474-11-208

**Published:** 2010-09-14

**Authors:** Akimoto Nimura, Takeshi Muneta, Koji Otabe, Hideyuki Koga, Young-Jin Ju, Tomoyuki Mochizuki, Koji Suzuki, Ichiro Sekiya

**Affiliations:** 1 Section of Orthopedic Surgery, Graduate School Tokyo Medical and Dental University, Tokyo, Japan; 2 Center of Excellence Program for Frontier Research on Molecular Destruction and Reconstruction of Tooth and Bone, Tokyo Medical and Dental University, Tokyo, Japan; 3 Section of Cartilage Regeneration, Graduate School, Tokyo Medical and Dental University, Tokyo, Japan; 4 Research & Development, JMS Company Limited, Hiroshima, Japan

## Abstract

**Background:**

Though sera are essential for Mesenchymal stem cells (MSCs), the effect of heat-inactivation remains unknown. Autologous human serum is recommended for clinical use; however, it is unclear whether differentiation potentials are maintained. To examine whether heat-inactivation of serum affected the proliferation and whether autologous human serum influenced their multipotentiality.

**Methods:**

After whole blood collection, human synovium and bone marrow were harvested. Nucleated cells were expanded with autologous human serum and FBS.

**Results:**

Heat-inactivation of autologous human serum enhanced proliferation of synovial MSCs. Heat-inactivation of each types of serum didn't affect calcification of synovial MSCs. The induction of calcification increased ALP activity, with the exception of bone marrow MSCs with autologous human serum. For adipogenesis of synovial MSCs, the Oil Red-O positive colony forming efficiency with autologous human serum was similar to or less than that with FBS.

**Conclusion:**

These clarified the processing of human autologous serum and the influence of different sera for differentiation of synovial and bone marrow MSCs.

## Background

Mesenchymal stem cells (MSCs) are useful for regenerative medicine because of their significant proliferation and multipotential abilities. Synovial MSCs are attractive for their high chondrogenic as well as calcification and adipogenic potentials [1,2]. Increasing the safety of medical treatments with MSCs requires the use of autologous human serum [3]. In our recent report, autologous human serum significantly increased the proliferation of synovial MSCs; in contrast, compared with fetal bovine serum (FBS), human serum decreased the proliferation of bone marrow MSCs [4].

Heat-inactivation of serum is known to reduce hemolytic and activation of complement components and to destroy some of the viruses and micro-organisms [5,6]. Heat-inactivation drastically changes protein contents [7]; however, the influences of heat-inactivation of human autologous serum as well as FBS on MSC proliferation and multipotentiality are hardly known.

Though the effect of autologous human serum on chondrogenic potential was examined [4], it is still unclear whether calcification and adipogenic potentials of human synovial and bone marrow MSCs were influenced by supplementation of autologous human serum. In this study, we first examined the effect of heat-inactivation of autologous human serum and FBS on proliferation of both populations of MSCs. Then, we compared the effect of autologous human serum and FBS on calcification and adipogenic potential of synovial and bone marrow MSCs. Our study will clarify the processing of human autologous serum and the influence of different serums for calcification and adipogenesis of synovial and bone marrow MSCs.

## Methods

### Tissue collection

The study was approved by the local institutional review board, and all human study subjects provided informed consent. Human synovium and bone marrow were harvested from 16 donors (25 ± 5 years, 14 males and 2 females) during the operation for anterior cruciate ligament reconstruction of the knee. Average disease duration was 14.1 months (range 1-65 months). Bone marrow from the tibia was obtained with an 18-gauge needle. Synovium with subsynovial tissue from the inner side of the medial joint capsule, which overlies the noncartilaginous areas of the medial condyles of the femur, was harvested with a pituitary rongeur under arthroscopic observation.

### Isolation of human serum

One day before the ligament reconstruction operation, 100 ml of whole blood was obtained from all donors using the closed bag system (JMS, Hiroshima, Japan) [4]. The system consists of a blood donation bag containing glass beads, which function by activating platelets and by removing fibrin from whole blood through a gentle mixing process for 30 minutes. After centrifugation at 2,000 *g *for 7 minutes, the serum was isolated. The serum was then filtered through a 0.45-μm nylon filter (Becton Dickinson, Franklin Lakes, NJ). Most of the serum was heat-inactivated at 56°C for 30 minutes and the remaining was spared without heat-inactivation. Both kinds were stored at -20°C until use.

### Isolation and expansion of MSCs

Synovium was digested in a solution of 3 mg/ml collagenase D (Roche Diagnostics, Mannheim, Germany) in Hanks' balanced salt solution (HBSS; Invitrogen, Carlsbad, CA) at 37°C. After 3 hours, the digested cells were filtered through a 70-μm nylon filter (Becton Dickinson). Nucleated cells from the bone marrow were isolated with a density gradient (Ficoll-Paque; Amersham Biosciences, Uppsala, Sweden). Nucleated cells were plated into 60-cm^2 ^dishes at clonal density, which was 10^3 ^or 10^4^/60-cm^2 ^dishes for synovial cells and 10^3 ^or 10^4^/cm^2 ^for bone marrow cells [1]. The cells were cultured in the complete culture medium (α-minimum essential medium [α-MEM; Invitrogen], 100 units/ml penicillin, 100 μg/ml streptomycin, and 250 ng/ml amphotericin B [Invitrogen]) containing 10% autologous human serum with heat-inactivation or 20% FBS without heat-inactivation for 14 days as passage 0.

### Heat-inactivation assay

Part of the FBS was heat-inactivated at 56°C for 30 minutes. To examine the effect on proliferation, passage 0 cells were plated at 50 cells/cm^2^, cultured for 14 days with 10% autologous human serum or 20% FBS, with or without heat-inactivation as passage 1, and harvested to count the cell number. To examine the effect on calcification of synovial MSCs, synovial MSCs precultured with autologous human serum or FBS at passage 0 were plated at 150-cm^2 ^dishes. These cells were cultured in the medium containing the same serum used at passage 0 for 14 days to form cell colonies. Then, the medium was switched into calcification medium containing the same serum used at passage 0 with or without heat-inactivation and cultured for an additional 21 days. The dishes were stained with Alizarin Red for calcification. The ratio of Alizarin Red positive colony number to the total colony number was calculated (n = 3).

### Calcification

One hundred cells acquired at passage 0 were plated in 150 cm^2 ^dishes and cultured for 14 days in complete culture medium containing 10% autologous human serum with heat-inactivation or 10% fetal bovine serum without heat-inactivation to produce cell colonies as passage 1. The medium was then switched to calcification medium that consisted of the complete medium with 10% autologous human serum with heat-inactivation or 10% FBS without heat-inactivation supplemented with 10^-9 ^M dexamethasone (Sigma-Aldrich, St. Louis, MO), 20 mM β-glycerol phosphate (Wako Pure Chemical Industries, Osaka, Japan), and 50 μg/ml ascorbate-2-phosphate for an additional 21 days. These dishes were stained with 0.5% Alizarin Red solution for 5 minutes, and the number of alizarin red-positive colonies was counted. The same calcification cultures were subsequently stained with 0.5% Crystal Violet in methanol for 5 minutes, and the number of total cell colonies was counted. Colonies less than 2 mm in diameter or yellowish colonies were ignored. The ratio of Alizarin Red-positive colonies to the total number of colonies was calculated for each dish.

### Alkaline phosphatase

MSCs acquired at passage 0 were plated at 5,000 cells/cm^2 ^in the complete culture medium with 10% autologous human serum with heat-inactivation or 10% FBS without heat-inactivation as passage 1. The next day the medium was switched to calcification medium or the complete culture medium. Ten days later, the cells were harvested with 1 ml lysis buffer (0.1 M Tris-HCl, 5 mM MgCl_2_, 2% Triton-X 100, 1 mM PMSF) and sonicated (Sonics & Materials, Newtown, CT). An aliquot (10 μl) of supernatant was added into 100 μl of 50 mM p-nitrophenylphosphatase hexahydrate containing 1 mM MgCl_2 _and the mixture was incubated at 37°C for 30 min. The absorption at 405 nm was measured with a spectrophotometer (Bio-Rad Laboratories, Hercules, CA). Data were normalized with the cellular protein content, and alkaline phosphatase activity was represented with mM of ρ-nitrophenol/mg protein release after 30 min of incubation at 37°C.

### Adipogenesis

One hundred cells acquired at passage 0 were plated per 60 cm^2 ^dish and cultured for 14 days in the complete culture medium with 10% autologous human serum with heat-inactivation or 10% FBS without heat-inactivation to make cell colonies as passage 1. The medium was then switched to adipogenic medium that consisted of complete medium with 10% autologous human serum with heat-inactivation or 10% FBS without heat-inactivation supplemented with 10^-7 ^M dexamethasone, 0.5 mM isobutylmethylxanthine (Sigma-Aldrich), and 50 μM indomethacin (Wako Pure Chemical Industries) for an additional 21 days. The adipogenic cultures were fixed in 4% paraformaldehyde, stained with fresh Oil Red-O solution, and the number of Oil Red-O-positive colonies was counted. Colonies less than 2 mm in diameter or faint colonies were ignored. The same adipogenic cultures were subsequently stained with Crystal Violet, and the number of total cell colonies was counted.

### Reverse transcription (RT)-PCR analysis

Total RNA was prepared by the RNAqueous Kit (Applied Biosystems, Foster City, CA) according to the manufacturer's instructions. RNA was converted to cDNA and amplified by the Instructions. Titan One Tube RT-PCR System (Roche Diagnostics). RT was performed by a 30-minute incubation at 50°C, followed by a 2-minute incubation at 94°C to inactivate the RT. PCR amplification of the resulting cDNAs was performed under the following conditions: 35 cycles of 94°C for 30 seconds, 58°C for 45 seconds, and 68°C for 45 seconds, in which the 68°C step was increased by 5 seconds every cycle after 10 cycles. The reaction products were resolved by electrophoresis on a 1.5% agarose gel and visualized with ethidium bromide. The PCR primers used are as follows:

Osteocalcin (forward) 5'-ATGAGAGCCCTCACACTCCTC-3',

Osteocalcin (reverse) 5'-GCCGTAGAAGCGCCGATAGGC-3' (297 bp),

PPARγ (forward) 5'-AAGACCACTCCCACTCCTTTG-3',

PPARγ (reverse) 5'-GTCAGCGGACTCTGGATTCA-3' (554 bp),

FABP4 (forward) 5'-ATGCTTTTGTAGGTACCTGG-3',

FABP4 (reverse) 5'-CTCTCTCATAAACTCTCGTG-3' (387 bp),

β-Actin (forward) 5'-CCAAGGCCAACCGCGAGAAGATGAC-3',

β-Actin (reverse) 5'-AGGGTACATGGTGGTGCCGCCAGAC-3' (587 bp).

### Statistical analysis

The Mann-Whitney *U *test was used for assessing differences. A value of P < 0.05 was considered to be statistically significant.

## Results

### Effect of heat-inactivation of serum on proliferation and calcification of human synovial and bone marrow MSCs

**Figure 1:**
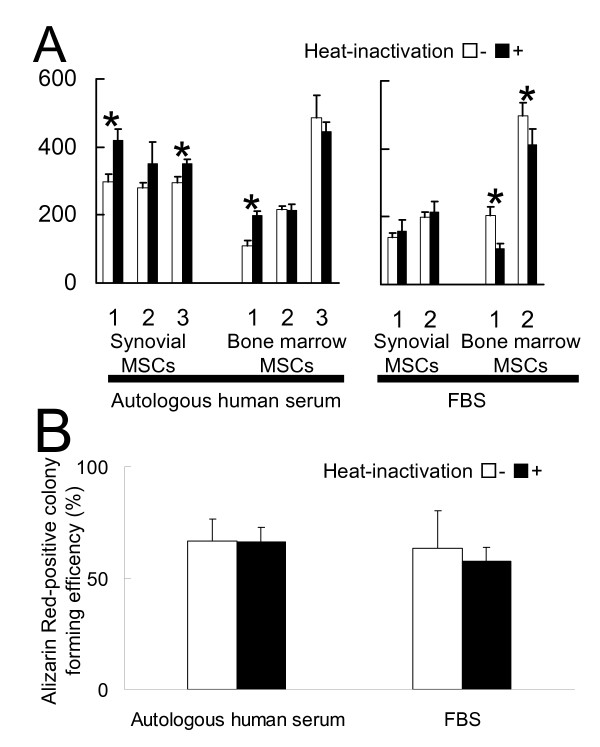
**Effect of heat-inactivation of serum on proliferation and calcification of human synovial and bone marrow MSCs**. (A) Synovial or bone marrow MSCs were plated at 50 cells/cm^2 ^and cultured in the presence of autologous human serum or FBS for 14 days. Two kinds of serum, one with or one without heat-inactivation, were used. Fold increases from each donor are shown respectively (n = 3). * = *P *< 0.01; fold increases in the same donor with the same serum with and without heat-inactivation. (B) Alizarin Red-positive colony forming efficiency of synovial MSCs cultured in the calcification medium containing the serum with and without heat-inactivation(%). Synovial MSCs precultured with autologous human serum or FBS at passage 0 were plated at 100 cells in 150-cm^2 ^dishes. These cells were cultured in the medium containing the same serum used at passage 0 for 14 days to form cell colonies. Then, the medium was switched into calcification medium containing the same serum used at passage 0 with or without heat-inactivation and cultured for an additional 21 days. The dishes were stained with Alizarin Red for calcification. The ratio of Alizarin Red positive colony number to the total colony number was calculated (n = 3). Average value with standard deviation from a donor is shown.

In synovial MSCs, heat-inactivation of autologous human serum enhanced the cell proliferation in 2 donors (Fig. 1A). Heat-inactivation of FBS seemed not to affect the cell proliferation. In bone marrow MSCs, the effects of heat-inactivation of autologous human serum varied in 3 donors. Heat-inactivation of FBS decreased the cell proliferation.

Heat-inactivation of each sera seemed not to affect the calcification of synovial MSCs (Fig. 1B).

### Calcification potential of human synovial and bone marrow MSCs cultured with autologous human serum or FBS

**Figure 2:**
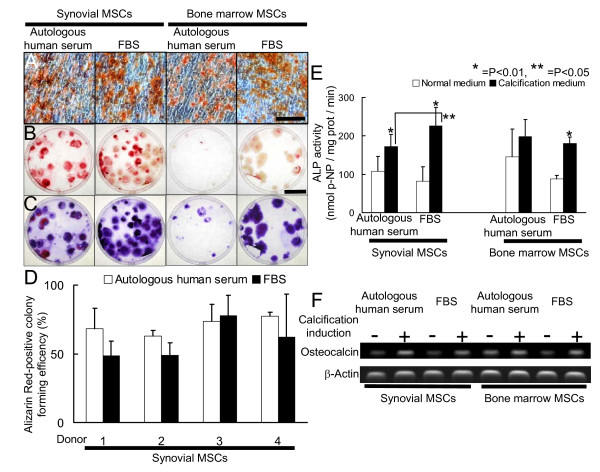
**Calcification potential of human synovial and bone marrow MSCs cultured with autologous human serum or FBS**. Human serum with heat-inactivation and FBS without heat-inactivation were used for further analyses. Synovial and bone marrow MSCs precultured with autologous human or FBS at passage 0 were replated at 100 cells in 150-cm^2 ^dishes. These cells were cultured in the medium containing the same serum used at passage 0 for 14 days to form cell colonies. Then, the medium was switched into calcification medium containing the same serum used at passage 0 and cultured for an additional 21 days. The dishes were stained with Alizarin Red for calcification. (A) Microscopic features of Alizarin Red-positive calcium nodule. Bar 100 μm. (B) Calcified cell colonies stained with Alizarin Red. Bar 5 cm. (C) Total cell colonies. The same dishes shown in (B) were stained with Crystal Violet. (D) Alizarin Red-positive colony forming efficiency of synovial MSCs (%). The ratio of Alizarin Red positive colony number to the total colony number was calculated (n = 3). Average value with standard deviation from 4 donors is shown respectively. (E) Alkaline phosphatase activity. Synovial or bone marrow MSCs at passage 0 were replated at 5000 cells/cm^2 ^and cultured in the absence or presence of calcification medium with autologous human serum or FBS for 10 days. Average value with standard deviation is shown (n = 3). (*P < 0.01 vs. control, **P < 0.05 vs. FBS) (F) Reverse-transcription-PCR analysis for osteocalcin. Synovial or bone marrow MSCs at passage 1 were replated at 5000 cells/cm^2 ^and cultured in the absence or presence of calcification medium with autologous human serum or FBS for 21 days.

Synovial and bone marrow MSCs calcified irrespective of serum (Fig. 2A). To quantify the calcification potential, the Alizarin Red positive colony forming efficiency was compared. In synovial MSCs, the serum seemed not to affect the Alizarin Red positive colony forming efficiency (Fig. 2B-D). In bone marrow MSCs, colony formation was hardly observed in culture with autologous human serum, which made it difficult to compare the efficiency of calcified-colony formation between the two serums, though autologous human serum appeared to reduce the Alizarin Red positive colony forming efficiency (Fig. 2A-C).

To deny the influence of colony formation, the cells were plated at a higher density, and alkaline phosphatase activity (ALP) was examined. The induction of calcification increased ALP activity, with the exception of bone marrow MSCs cultured with autologous human serum due to their high activity of ALP in the uninduced cultures (Fig. 2E). In synovial MSCs induced into calcification, FBS enhanced ALP activity in comparison to autologous human serum.

Calcification induction increased osteocalcin mRNA expression in both synovial and bone marrow MSCs irrespective of serum (Fig. 2F). Without calcification induction, osteocalcin mRNA expression in bone marrow MSCs with autologous human serum was higher than that with FBS, as shown in ALP activity.

### Adipogenic potential of human synovial and bone marrow MSCs cultured with autologous human serum or FBS

**Figure 3:**
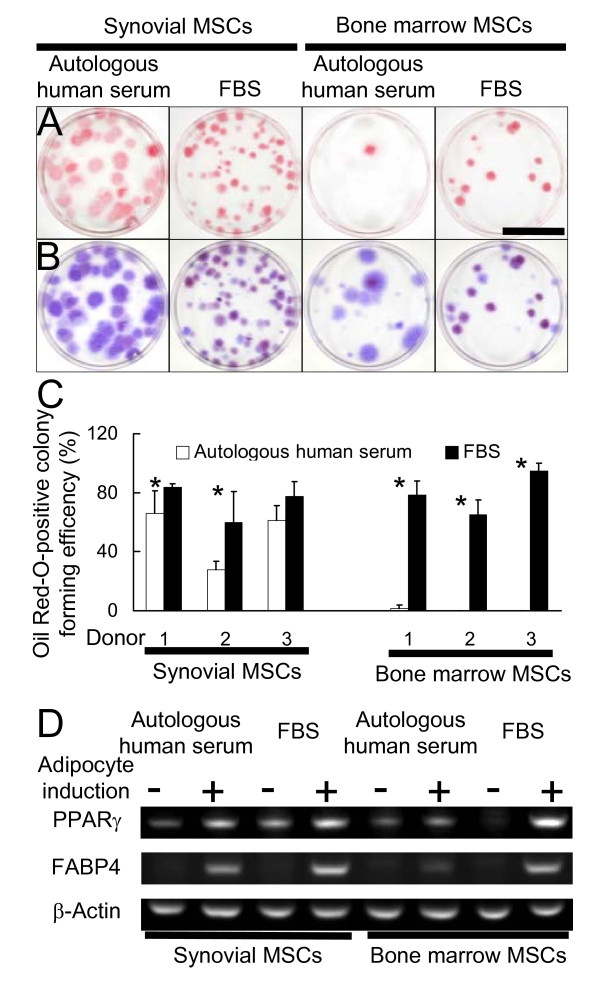
**Adipogenic potential of human synovial or bone marrow MSCs cultured with autologous human serum or FBS**. Synovial MSCs and bone marrow MSCs precultured with autologous human serum or FBS at passage 0 were replated at 100 cells in 60-cm^2 ^dishes. These cells were cultured in the medium containing the same serum used at passage 0 for 14 days to form cell colonies. Then, the medium was switched into adipogenic medium containing the same serum used at passage 1 and cultured for an additional 21 days. The dishes were stained with Oil Red-O for adipogenesis. (A) Adipocyte colonies stained with Oil Red-O. Bar 5 cm. (B) Total cell colonies. The same dishes shown in (A) were stained with Crystal Violet. (C) Oil Red-O positive colony forming efficiency (%). The ratio of Oil Red-O positive colony number to the total colony number was calculated in synovial and bone marrow MSCs (n = 3). Average value with standard deviation from 3 donors is shown respectively. * = *P *< 0.01; Oil Red-O positive colony forming efficiency in the same donor with and without adipocyte induction. (D) Adipocyte-related mRNA expressions by reverse transcription-PCR analysis. Synovial or bone marrow MSCs at passage 0 were replated at 5000 cells/cm^2 ^and cultured in the absence or presence of calcification medium with autologous human serum or FBS for 21 days. PPARγ indicates peroxisome proliferator activated receptor γ; and FABP4, fatty acid binding protein 4.

In synovial MSCs, the Oil Red-O positive colony forming efficiency with autologous human serum was similar to or less than that with FBS. In bone marrow MSCs, autologous human serum dramatically decreased the Oil Red-O positive colony forming efficiency in comparison to FBS (Fig. 3A-C). RT-PCR also demonstrated that autologous human serum reduced mRNA expressions for peroxisome proliferator activated receptor γ(PPARγ) and fatty acid binding protein 4 (FABP4), specific genes for adipogenesis, in bone marrow MSCs cultured in adipogenic medium (Fig. 3D).

## Discussion

There have been only a few studies describing heat-inactivation of human serum and FBS for bone- and bone marrow- derived cells. Hankey reported that heat-inactivation of autologous human serum increased the growth of osteoblasts and FBS did not [8]. Bruinink showed that heat-inactivation of human serum decreased the growth of bone marrow MSCs in contrast to that of FBS which increased it [9]. In our current study, heat-inactivation of autologous human serum did not affect the growth of bone marrow MSCs, but that of FBS decreased proliferation of bone marrow MSCs. These three reports showed totally different results for bone- and bone marrow- derived cells.

We showed that heat-inactivation of autologous human serum increased expansion of synovial MSCs. As possible mechanisms, heat-inactivation reduces anti-complementary activity [8] and may raise the concentration of some mitogenic factors [7]. We recommend heat-inactivate autologous human serum for expansion of synovial MSCs. When cultured in calcification medium, autologous human serum increased ALP activity and osteocalcin mRNA expression of synovial MSCs in comparison with that of the control medium but did not affect bone marrow MSCs.

Interestingly, when cultured in control medium, autologous human serum increased ALP activity and osteocalcin mRNA expression of bone marrow MSCs in comparison with that of FBS. Oreffo et al. [10], Hankey et al. [8], and Stute et al. [11] also reported that autologous human serum promoted ALP activity and von Kossa staining of bone marrow MSCs more than FBS did. In contrast, the calcification potential of synovial MSCs was not affected by the two different serums. Certain factors inducing calcification of bone marrow MSCs should exist in human serum.

For adipogenesis of bone marrow MSCs, autologous human serum markedly reduced adipogenic potential. Human serum contains a high concentration of platelet derived growth factor (PDGF), which is known to have an inhibitory effect on human adipocyte differentiation, and this may have caused the reduced adipogenic potential in bone marrow MSCs. Artemenko et al. described how PDGF-BB suppressed triglyceride accumulation and expression of adipogenic specific transcriptional factors during 3T3-L1 preadipocyte differentiation [12,13]. Stute et al. also showed that bone marrow MSCs cultured with autologous human serum inhibited adipogenic differentiation in comparison with those with FBS [11]. However, Oreffo et al. reported that bone marrow MSCs cultured with human serum induced more adipogenesis than those with FBS after induction with dexamethasone [10]. Responsiveness of the two serums for adipogenesis of bone marrow MSCs depends on agents which were included in the medium for the differentiation.

The two different serums hardly affected adipogenic potential of synovial MSCs, the results being totally different from the case of bone marrow MSCs. This may raise an interesting conundrum. We previously demonstrated that human serum is rich in PDGF, especially PDGF- AA and AB; however, PDGF receptor α, which binds to PDGF-AA and AB, is expressed more in the synovial MSCs than in bone marrow MSCs [4]. Different expression of PDGF receptor α might have affected the results.

This study has some limitations that could restrict the generalization of our findings. First, we collected the data from only young donors for our research. There is concern that age of donors effects the potential of cultured cells. If synovial MSC implantation can be indicated for older individuals with osteoarthritis in the future, we can better further analyze the effect of human autologous serum from older individuals. Second, for isolation and expansion of MSCs, 10% autologous human serum and 20% FBS were used. For our preliminary study, 10% FBS was used, but we could not obtain a sufficient number of synovial MSCs for further analysis; therefore, we increased the concentration of FBS to 20%. Due to the limited availability of human serum, the concentration of human serum was kept at 10%. Finally, for calcification and adipogenesis analysis, autologous human serum with heat-inactivation and FBS without heat-inactivation was compared. Based on the results of Fig. 1A, autologous human serum with heat-inactivation and FBS without heat-inactivation were preferred from the viewpoint of cell yield. Therefore, from the standpoint of practical situations, these changes were supplemented with analyses of calcification and adipogenesis. So the effects of heat-inactivation on calcification and adipogenesis of MSCs remain uncertain.

## Conclusion

Heat-inactivation of autologous human serum enhanced expansion of synovial MSCs but did not affect bone marrow MSCs. ALP activity of synovial MSCs cultured in calcification medium was increased when autologous human serum was used. Adipogenesis of bone marrow MSCs decreased when cultured with autologous human serum.

## List of abbreviations

MSCs: Mesenchymal stem cells; FBS: fetal bovine serum; RT: Reverse transcription; ALP: alkaline phosphatase activity; PPARγ: peroxisome proliferator activated receptor γ; FABP4: fatty acid binding protein 4; PDGF: platelet derived growth factor

## Competing interests

The authors declare that they have no competing interests.

## Authors' contributions

AN carried out all the assays and drafted the manuscript. TM and IS conceived of the study, and participated in its design and coordination. KO carried out the calcification assay. HK, YJ, and TM carried out the tissue collections. KS participated in the harvesting of human sera. All the authors read and approved the final manuscript.

## Pre-publication history

The pre-publication history for this paper can be accessed here:

http://www.biomedcentral.com/1471-2474/11/208/prepub
